# Editorial: Regenerating the dentin-pulp complex: understanding the challenges that lie ahead

**DOI:** 10.3389/fbioe.2023.1249969

**Published:** 2023-07-11

**Authors:** Rania El Backly, Samer Zaky, Mona K. Marei

**Affiliations:** ^1^ Endodontics, Conservative Dentistry Department, Faculty of Dentistry, Alexandria University, Alexandria, Egypt; ^2^ Tissue Engineering Laboratories, Faculty of Dentistry, Alexandria University, Alexandria, Egypt; ^3^ School of Dental Medicine, University of Pittsburgh, Pittsburgh, PA, United States; ^4^ Prosthodontics Department, Faculty of Dentistry, Alexandria University, Alexandria, Egypt

**Keywords:** dentin-pulp complex regeneration, tissue engineering & regenerative medicine, dental pulp-derived cells, bone engineering, corneal tissue, regenerative endodontics

The field of endodontic tissue regeneration has been rapidly gaining momentum over the past two decades (Elnawam et al., 2022). The notion that an entire new functional dentin-pulp complex can be regenerated has been a quest for dental researchers since the discovery of post-natal dental pulp stem cells in the early 2000s (Gronthos et al., 2000). However, since coining of “regenerative endodontics” in the seminal article by Peter Murray et al., in 2007, the dream has been catapulted into everyday clinical practice (Murray et al., 2007). This Research Topic focused on including research articles that not only highlight novel strategies to regenerate the dental pulp but also articles that provide insight on the robust capacities and potential of dental pulp derived cells for the regeneration of non-dental tissues.

It is safe to say that among all the applications of regenerative medicine, revitalization or revascularization procedures may represent the simplest form of a cell-homing strategy towards tissue regeneration that has really found its way into the hands of clinicians (Astudillo-Ortiz et al., 2023). Interestingly, the realization that the dentin-pulp complex does have some capacity to repair/regenerate is not new. The use of calcium hydroxide for vital pulp therapy procedures has repeatedly demonstrated that new tertiary dentin matrix can be deposited in response to reactionary or reparative dentinogenesis. This phenomenon has been even more elucidated in response to recently developed calcium silicate or bioceramic materials. Further studies have shown that the mechanisms and pathways involved in the trigger of reparative dentinogenesis do involve the recruitment of viable dental pulp stem/stromal or progenitor cells from the remaining inflamed or healthy pulp tissue. These materials have demonstrated the ability to modulate the inflammatory response within the pulp tissue to a great extent thereby favoring repair/regeneration. This has led to more intricate studies attempting the understanding of the developmental mechanisms behind these events to better target the stem cell niche thereby targeting more site-specific tissue regeneration.

On that note, Fu et al. venture into a study employing decellularized dental pulp extracellular matrix (ECM) to regenerate the dental pulp. As many growth factors and chemokines key for cellular differentiation have been reported to wash out or at least remain in traces during the decellularization process (Song et al., 2017; Alqahtani et al., 2018), a next-generation of ECM application research is investigating if the addition of the lost factors post-decellularization would rescue its native biological function. This time, Fu et al. enhanced their biological scaffold with laminin supplementation which, using an *in vivo* model, reportedly improved homing cell adhesion within the ECM and histologically showed neovascularization and a properly oriented layer of odontoblast-like cells with regenerative dentin.

As longer-term evidence in the field of endodontic regeneration becomes available, complications and challenges are becoming more apparent, emphasizing the need for optimizing protocols to enhance treatment outcome predictability in both research and clinical settings. Is new vascularized pulp tissue possible to regenerate? Can tubular dentin be regenerated with the same morphological features of primary dentin? How can the dental pulp stem cell niche be targeted and recreated? These and many other questions are only some of the challenges facing the advancement of the field of endodontic regeneration. Indeed, Ruan et al. review the challenging task of prevascularizing a dental pulp engineering construct. Parallel to our ascending curve of understanding dental pulp biology, such techniques while still in their infancy, are gaining ground as different strategies are investigating the most applicable cell sources for optimum intercellular communication. The review also tackles innovative ideas and techniques to boost the vascularization of tissues for dental pulp regeneration.

Another interesting aspect in the study of dentin/pulp complex regeneration is that as the mechanisms of repair/regeneration are further investigated, immense new potentials of pulp derived cells are being discovered everyday further supporting the interdisciplinary application of these cells for other therapies. While these cellular populations harvested from the dental pulp are intuitively and primarily used to research dental pulp regeneration, their readiness of availability (from exfoliated deciduous teeth (SHED) and from routine third molar extraction) encourages their investigation in ectopic applications. In that aspect, Xiong et al. report that for bone regeneration applications, employing a bioactive glass scaffold enhanced the osteogenic potential of SHED mechanistically by enhancing the activation of AMP kinase signaling. The construct is reportedly promising for critical size defect injuries as bone formation was evidently enhanced in the center of the *in vivo* defect.

Another application for dental pulp cells is reported here by Zhu et al. where they thoroughly characterized cryopreserved dental pulp cells that, upon recovery, could be readily employed to enhance bone tissue engineering constructs and be reliably useful as a source of osteogenic cells for delayed therapies. Furthermore, and diverging from the same embryonic origin, dental pulp cells have also been reported to be an auspicious substitute for corneal endothelial cells. Under this Research Topic, Bosh et al. confirm (Syed-Picard et al., 2018) that patient-derived dental pulp stem cells, bypassing transplantation limitations as well as reprogramming techniques, represent a handy autologous cell source for corneal endothelial therapies.

It remains to be said that there is still an immense gap between basic research and clinical translation in the field of endodontic regeneration with a wide disparity in approaches and techniques aiming to regenerate the dentin/pulp complex. Perhaps this reflects the bigger conflict in the wider field of tissue engineering and regenerative medicine, supported by advocates of cell-based transplantation approaches for tissue regeneration with the belief that true biological and functional tissue can only be regenerated using cell-based therapies. On the other hand, the only way for clinical translation to move forward is by offering affordable, reproducible, chair side methods to enhance cell-homing strategies for tissue regeneration. It could be possible that the only way for these fields to move forward is by not focusing on the semantics of repair or regeneration but rather focus on a hierarchical approach to reach a final goal of regeneration. This would be a translational path starting with the simpler replacement of tissue going on to repair and finally reaching the so called “holy grail” of complete tissue regeneration. At the same time, these goals should coincide with clinician, patient, and finally scientist-centered outcomes with the hope of someday reaching the regeneration of a truly functional dentin-pulp complex. While this approach is being followed, it must also coincide with the objectives of developing personalized therapies that are patient-customized taking into respect not only the genetics but also epigenetic regulation of tissue repair and regeneration.
